# Superior forward osmosis cellulosic membrane for water desalination and brine concentration

**DOI:** 10.1038/s41598-025-00141-z

**Published:** 2025-05-14

**Authors:** Rania Sabry, Hanaa M. Ali, Sahar S. Ali, Hanaa Gadallah

**Affiliations:** Chemical Engineering Department, Engineering and Renewable Energy Research Institute, National Research Centre, 33 El-Bohouth St. (Former El-Tahrir St.), Dokki, PO Box 12622, Giza, Egypt

**Keywords:** Forward osmosis, Cellulose acetate, Polymer blend, Saline water desalination, Brine concentration, Environmental sciences, Chemistry, Engineering

## Abstract

Brine is an inevitable product of desalination plants and various industrial sectors. The appropriate disposal of brines and their highly concentrated salts and other diverse contaminants is a critical environmental challenge to most plants. Recently, the FO process is gaining attention as a viable alternative to conventional brine concentration methods, primarily due to its reduced energy consumption. This study aimed to fabricate a high-performance nonwoven FO membrane from cellulose acetate (CA) blended with polysulfone (PS) via the phase inversion technique. The prepared membrane was investigated in both saline water desalination and brine concentration. The FO membrane performance was tested for saline water desalination at different NaCl concentrations and applied in brine concentration using two kinds of draw solutions: ammonium bicarbonate (NH_4_HCO_3_) and a mixture of ammonium hydroxide/ammonium bicarbonate (NH_4_ OH)/(NH_4_HCO_3_) at different concentrations. It was observed that a high flux of 150 LMH was attained by using 2 M NaCl as a draw solution against distillate water as a feed solution. For brine concentration, by application of (NH_4_ OH)/(NH_4_HCO_3_) draw solution mixture at a 1:1 ratio, the modified cellulosic FO membrane exhibited the highest water flux of 113 LMH.

## Introduction

The escalating global demand for water has significantly accelerated the growth of desalination plants, which are essential for meeting the needs of various intensive human activities^1–3^. Recent estimates indicate that in 2022, there were over 21,000 seawater desalination plants worldwide, collectively producing 99 million cubic meters of desalinated water per day, while also generating more than 150 million cubic meters of brine byproduct daily^4^.

The environmentally adverse impact of brine arises from its salinity (1.6–2 times greater than seawater salinity (35 g/L)), temperature, and chemical composition. The temperature of brine discharged from membrane desalination systems is at ambient seawater temperature (22°C), whereas the brine from thermal desalination processes is generated at a temperature 1.37–1.82 times greater than 22°C^5,6^. The primary environmental concerns associated with brine disposal include increased salinity of nearby water bodies and soil, the impact of elevated brine salinity on marine benthic communities near the disposal site, aesthetic deterioration, the disposal of pretreatment and membrane cleaning chemicals, and corrosive metals^7^.

Many investigations indicate that even a minor rise in salinity can adversely affect marine organisms by disturbing their osmotic balance with the environment^8–13^. This disruption results in cellular dehydration, a reduction in turgor pressure, and may ultimately contribute to species extinction over time^14,15^. Petersen et al. recently noted that elevated salinity (10% over ambient levels) adversely affected the physiological and visual characteristics of the coral. The elevated salinity, along with the introduction of polyphosphate-based anti-scalants, significantly affected all examined coral species^16^. Consequently, safe disposal of brine is the major technical and economical challenge, and regulatory bodies have initiated strict sets of measures to regulate the treatment of brines prior to discharge^17,18^.

Traditional methods for managing brine include surface water discharge^19^, sewer disposal^20^, deep-well injection^21^, evaporation ponds, and land application^7,22^. These approaches are typically associated with high capital expenditures and adverse environmental impacts, or sometimes both. Conversely, environmental and economic issues about brine discharge, along with sustainability demands for enhanced water recovery in desalination processes, have intensified the urgent requirement for more economical and sustainable alternatives^23^. Recently, brine treatment has increasingly adopted a minimum or zero-liquid discharge (M/ZLD) strategy, which focuses on water reclamation and minimizing waste volumes^24^.

Advanced M/ZLD is accomplished by evaporative brine concentrators (EBC) and crystallizers (EBCr). Nonetheless, both EBCs and EBCrs are excessively capital and energy-intensive for widespread application, particularly at flow rates below around 500 m^3^/day^25^. The specific energy consumption (SEC) of these procedures varies from approximately 20 kWh/m^3^ (EBC) to more than 100 kWh/m^3^ (EBCr)^25,26^. Although the majority of this energy is thermal and relatively inexpensive, it is predominantly sourced from fossil fuel consumption, resulting in the emission of both primary air pollutants and CO_2_. Membrane and thermal technologies can be utilized to concentrate brine and provide optimal water recovery, an approach referred to as zero liquid discharge (ZLD)^27^. These may encompass pressure-driven osmosis processes^28^, membrane distillation^29^, electrodialysis^30^, forward osmosis concentrators and crystallizers^31^, multi-stage flash distillation, and multi-effect distillation^32^.

The forward osmosis (FO) process, an innovative membrane technology, has garnered significant interest from both the research community and industry. The FO process operates on the principle of using osmotic pressure to drive water through a semipermeable membrane from a diluted feed solution (FS) to a more concentrated draw solution (DS)^24^. The osmotic pressure difference between the two sides of the membrane serves as the driving force for water transport.

For the first time, an NH_3_/CO_2_ draw solution was utilized in a pilot-scale forward osmosis treatment facility by Oasys Water in Boston, USA, to concentrate generated water utilizing a polyamide thin film composite membrane^33^. Highly saline generated water (70,000 mg/L) was concentrated to 180,000–200,000 mg/L by the pilot plant, achieving a 60% recovery of product water with less than 500 mg/L total dissolved solids (TDS), which complies with the allowable limits for surface water discharge^33^.

In China, FO process concentrated a blend of wastewater and cooling tower blowdown at a feed rate of 650 m^3^/day, yielding makeup boiler feed water with TDS under 100 mg/L^34^. This configuration initiates with RO to elevate feed water TDS to around 60,000 mg/L, succeeded by FO utilizing the NH_3_/CO_2_ draw solution to increase the RO concentrate to over 220,000 mg/L TDS. The process concludes with the FO concentrate being directed to a crystallizer, which concurrently generates high-quality product water (TDS < 100 mg/L post-secondary RO treatment) appropriate for reuse as boiler makeup water.

Recent advancements have spotlighted FO for brine concentration applications. Panagopoulos et al. (2019) evaluated FO’s efficiency in extracting water from brine and compared it with other Zero Liquid Discharge (ZLD) membrane technologies, including Reverse Osmosis (RO), High-Pressure RO (HPRO), and Osmotically Assisted RO (OARO)^7^. Their findings indicated that FO is more cost- effective for high-salinity brines (≤ 200 g/L). Other studies^35–37^ demonstrated that FO can concentrate brine from natural gas extraction to levels between 73 and 180 g/L using an NH_3_/CO_2_ FO membrane concentrator^37^.

The evolution of FO technology has led to significant enhancements in process performance^33,38^. Key advancements involve optimizing draw solutions and developing high-efficiency FO membranes^38^. FO-specific membranes are typically classified into Cellulose Tri-acetate (CTA), Cellulose acetate/Cellulose Tri- acetate (CA/CTA), Thin film composite (TFC), and biomimetic membranes, with chemically modified membranes also being widely utilized^39,40^. CTA-FO membranes are favored in wastewater treatment due to their chlorine tolerance, resistance to biodegradation, hydrophilicity, and low fouling propensity^41–43^.

CA is of significant interest to researchers because to its biodegradable characteristics and hydrophilic nature, which allow for functionalization with various groups to attain desired qualities^44,45^. The CA membranes exhibit high chemical and mechanical durability, exceptional transport capabilities, minimal protein adsorption, excellent water affinity, low toxicity, superior film-forming capability, and easy accessibility^46^.

However, CTA membranes face limitations such as pH and temperature instability and low water permeability^47,48^. Conversely, thin film composite (TFC) membranes offer superior performance due to their broad pH tolerance (2-23), stability at temperatures over 60°C, and enhanced water permeability^49^. Despite these advantages, TFC membranes are susceptible to fouling and concentration polarization, impacting operational costs and requiring adjustments in the structural parameter of the FO sub-layer (S value)^50,51^. The two-stage manufacturing process also elevates costs, and the polyamide active layer may be vulnerable to chlorine^42^.

Recent efforts have focused on developing CA FO membranes with improved properties through two primary modification methods: surface modification (e.g., plasma treatment, surfactant modification, coating, UV irradiation, interfacial polymerization)^50^ and blending modification (modifying polymer matrices before membrane formation). The latter method enhances polymer processing, material selection, and membrane hydrophilicity^52,53^. El-Ghaffar et al. indicated that combining of several polymers yields advantages including increased membrane hydrophilicity, greater physicochemical stability, superior polymer film-forming attributes, and improved antifouling capabilities^54^. Investigations into new materials for FO membrane fabrication aim to boost water flux, reduce ICP, and improve water quality tolerance, often by blending natural and synthetic polymers^55^.

Polysulfone (PS) is a preferred option in membrane development due to its availability, ease of modification, chemical stability, high mechanical strength, surface charge, and broad operational temperature and pH range^56^. PS has alkyl or aryl sulfone chemical groups, resulting in minimal cytotoxicity and inherent biocompatibility^57^. The remarkable biocompatibility of PS is attributed to its intrinsic high concentration of aromatic groups^58^. PS is widely regarded as an appropriate material for wastewater treatment membranes due to its superior mechanical properties, with a tensile strength ranging from 70 to 83 MPa^59^.

Yuan et al. synthesized a homogenous PS nanofiltration membrane with a compact structure using the solvent evaporation technique and employed an elevated membrane formation temperature (110°C) to enhance membrane performance^60^. The membrane was subsequently utilized to segregate Na_2_SO_4_ from sodium chloride (NaCl) in various mixed salt solutions. Additionally, the membrane’s resistance to chlorine, thermal stability, and mechanical strength were assessed. The findings demonstrated that the membrane exhibited substantial mechanical strength exceeding 66 MPa and exceptional chlorine resistance. It maintained effective separation capability despite exposure to a 50,000 mg/L sodium hypochlorite solution for 24 h.

Syahbanu et al. fabricated an ultrafiltration PS/CA blend membrane with a 15/5 (%wt.) ratio using the phase inversion method and examined the influence of casting evaporation duration on filtration efficacy^61^. The authors reported that the maximum water flux occurred at 2 bar, reaching 106.31 LMH at time zero, then diminished as evaporation time progressed. The morphology of PS/CA blend membranes indicated the formation of porous asymmetric membranes.

Douna et al. employed the solution casting technique to fabricate CA/PS composite membranes with varying concentrations of PS for the separation of CO_2_ and H_2_^62^. The gas permeation data indicate that CO_2_ permeability rises with higher concentrations of PS. Significant permeability (P = 80.51 Bar) of CO_2_ and a selectivity of CO_2_/H_2_ = 1.83 for CA/PS 2 wt% were attained at 25°C and 2.5 bar in comparison to the pure CA membrane.

This study aims to develop a cellulosic FO membrane with enhanced properties using a blending modification technique for saline water desalination and brine concentration. The membrane was produced by combining cellulose acetate with polysulfone in different proportions. The resultant membranes were characterized through scanning electron microscopy (SEM), Fourier-transform infrared spectroscopy (FTIR), water uptake rate, porosity, contact angle, and tensile strength. The FO membranes were evaluated for desalination efficacy at varying NaCl concentrations, and the ideal membrane was utilized for brine concentration employing ammonium bicarbonate and a combination of ammonium hydroxide/ammonium bicarbonate at varied concentrations. Finally, the morphology of the most effective FO membrane was examined after application to assess its fouling tendency in the brine concentration process.

To our awareness, this blend has not yet been investigated for forward osmosis applications.

## Materials and methods

### Materials

Cellulose acetate (CA) and polyethylene glycol (400) were obtained from Sigma Aldrich. The solvent N-methyl-2-pyrrolidone (NMP) was also manufactured by Sigma Aldrich. Polysulfone (PS) Udel P-3500 (PS, Mn: 22,000 Da) was purchased from Solvay Advanced Polymers (Alpharetta, GA, USA). Sodium chloride, ammonium bicarbonate, and ammonium hydroxide were purchased from Sigma Aldrich. Nonwoven fabric from China. And, commercial sodium chloride for synthetic brine preparation.

### Methods

#### FO membrane fabrication

Membrane blend solutions consisting of cellulose acetate (CA) and polysulfone (PS) were formulated at a total polymer concentration of 25 wt.%. The two polymers were dissolved in varying ratios, with 1% polyethylene glycol (PEG 400) included as an additive and 74% NMP as solvent. The mixtures were subjected to continuous mechanical stirring at a moderate rotational speed in a round-bottom flask for 3 h at room temperature. Following this, the homogeneous solutions were allowed to rest for a minimum of 12 h in an airtight environment to facilitate the removal of air bubbles. This preparation technique mirrors the “phase inversion” method utilized in previous studies as reported by other researchers^63^.

The homogenous polymer blending solutions contained CA and PS with different compositions (CA/PS: 100/0, 90/10, and 80/20). The solutions were cast on a clean glass plate with a fixed nonwoven fabric at a thickness of 85 µm, and the thickness of the membranes height over the fabric was maintained at 50 µm. The cast membranes were promptly immersed in an ice water coagulation bath, where they were maintained for 24 h to ensure thorough precipitation. Finally, the membranes were rinsed with DI water and preserved in a mixture of 0.1% formalin solution and 30% glycerol solution before characterization.

#### FO membrane characterization

##### FTIR spectroscopy

The chemical structure of the prepared nonwoven cellulose acetate and nonwoven CA/PS blend FO membrane was confirmed by the FTIR model JASCO FTIR-6100 spectrophotometer.

##### Membrane physical properties

FO membrane physical properties include water uptake rate, porosity, tortuosity (τ), structural parameters, and contact angle, outlined as follows:

(i) Membrane surface morphology: The prepared flat sheet membrane polymer blend is examined by Scanning Electron Microscope (SEM) which is used to observe the microstructures of the membranes prepared. In order to get morphology, the nascent membrane sheet must be dried and fractured in liquid nitrogen. Then, sputtering process with gold will be applied on the sheet to provide electrical conductivity, using SEM model JEOL 5410 which was operating of 10–30 kV.
(ii) The water contact angles of the prepared membranes were analyzed using the sessile drop technique and measured using a contact angle device (SL200C, SoLun)^48^.
(iii) The synthesized FO membranes were tested for water uptake rate and porosity by quantifying the change in weight before and after hydration. The membrane was firstly drenched in deionized water at room temperature for 24 h. The weight of the wetted membrane (Mw) was measured after wiping the surface using filter paper. The dried membrane’s weight (Md) was measured after drying the membrane in an air-circulating furnace at 80°C for 24 h. The percentage of water uptake was calculated by using the following equation^64^.
  1 $$\% Water\;uptake\;rate = \left( {M_{W} - M_{d} } \right)/M_{d} *100$$
(iv) The porosity of membranes (P) was calculated using the following equation^65^:
  2 $$P\% = \left( {M_{W} - M_{d} } \right)/V*100$$
  where P is the membrane porosity, Mw and Md are the weights of wet and dry membranes (g), respectively, and V = A*d, where A is the membrane surface area (cm^2^) and d is the membrane thickness (cm). Membrane tortuosity (τ) was calculated by using Eq. (3)^66^:
  3 $$\tau = \left( {2 - P} \right)^2/P$$
(v) The membrane’s structural parameters (S) were calculated by using Eq. (4)^67^.
  4 $$S\;\left( {\upmu {\text{m}}} \right) = \left( {d*\tau } \right)/p$$
(vi) Tensile strength: The tensile strength of the synthesized membranes was evaluated using a Hounsfield H10KS tensile testing machine, operating at a crosshead speed of 12.5 mm/min, under conditions of 75% relative humidity and a temperature of 27°C. The dimensions of the test samples were standardized at 5 mm in width and 50 mm in length. For each sample, three individual specimens were subjected to testing to calculate the average tensile strength and the percentage elongation at break.

#### Membrane performance

**Fig. 1:**
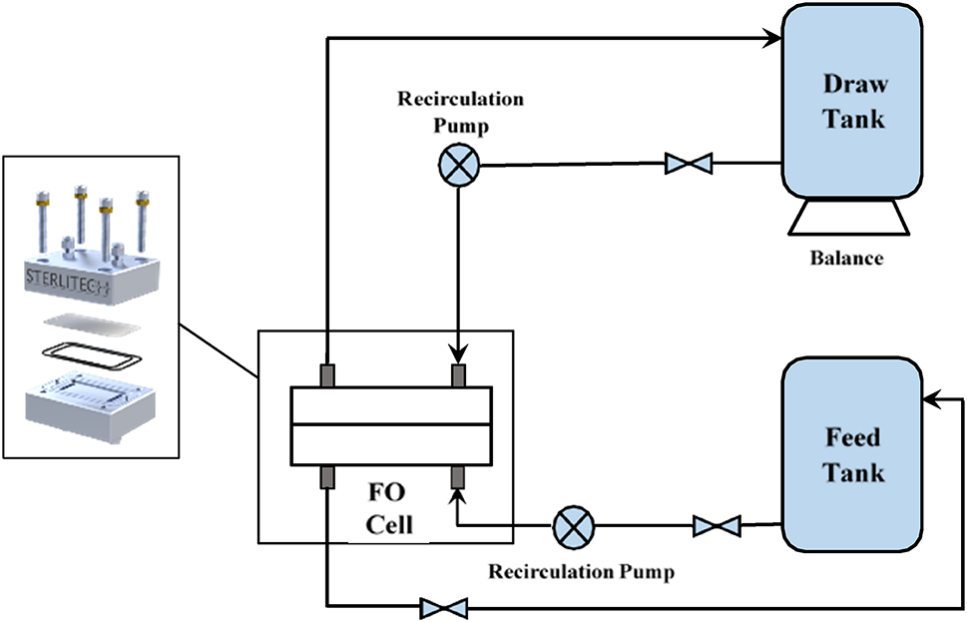
Bench-scale forward osmosis experimental setup.

The prepared polymer blend membranes were tested using Sterlitech’s FO bench scale test cell (CFO42). The system, as shown in Fig. 1, contains one membrane cell divided into two parallel channels; the outer dimensions of the cell are 12.7 × 10 × 8.3, and the active area dimension is 9.2 × 4.572. The effective surface area of the membrane is 42 cm^2^.

The flow of the two solutions (draw and feed) was designed to be counter- current flow on both sides of the membrane. The solutions flow was controlled independently by a gear pump (81808, Cole-Parmer, USA) and controlled at a rate of 0.857 L min^−1^. The DS tank was located on an analytical balance (XP8002S, Mettler Toledo, USA), and the rate of change of the DS weight was recorded. The total dissolved solid was measured by using a Myron L Ultrameter conductivity meter.

The configuration of the membrane was designated as the feed solution faced the active layer^67^. The membrane performance was determined during a 1-h experiment by determining the permeate flux (Jw) and reverse salt flux (Js).

The performance of the blend membrane was conducted using 1 M NaCl as the draw solution and distilled water as the feed solution. Ten minutes was chosen for stabilizing the system, after which the permeate flux was calculated by measuring the DS weight change with time using the subsequent equation (5)^68^

5 $$J_{w} = \Delta V/(A*\Delta t)$$

where Jw is water flux (LMH), ΔV is the permeate volume (L) that passes through the membrane during the time interval Δt (h), and A (m2) is the effective membrane area. The reverse salt flux (Js, g/m^2^ h) was determined by Eq. (6)^69^:

6 $$Js = \left( {C_{f} V_{f} - C_{0} V_{0} } \right)/A*\Delta t$$

where C_f_ and C_0_ (mol/L) are the final and initial salt concentration of the FS, V_f_ and V_0_ (L) are the final and initial volume of the FS.

#### Application for brine concentration

The performance of the modified FO membrane prepared at the optimum polymer blend ratio as a brine concentrator was studied by using two kinds of draw solutions: ammonium bicarbonate and a mixture of ammonium hydroxide/ammonium bicarbonate at different concentrations. The feed solution was a synthetic brine consisting of a commercial NaCl solution at a concentration of 65 mg/L. The performance was measured in terms of water fluxes across the membrane.

In addition, the fouling tendency of the selected FO membrane was investigated at optimum DS concentration by studying the flux against time at constant driving force across the membrane.

## Results and discussions

### Membrane characterization

#### FTIR spectroscopy

**Fig. 2:**
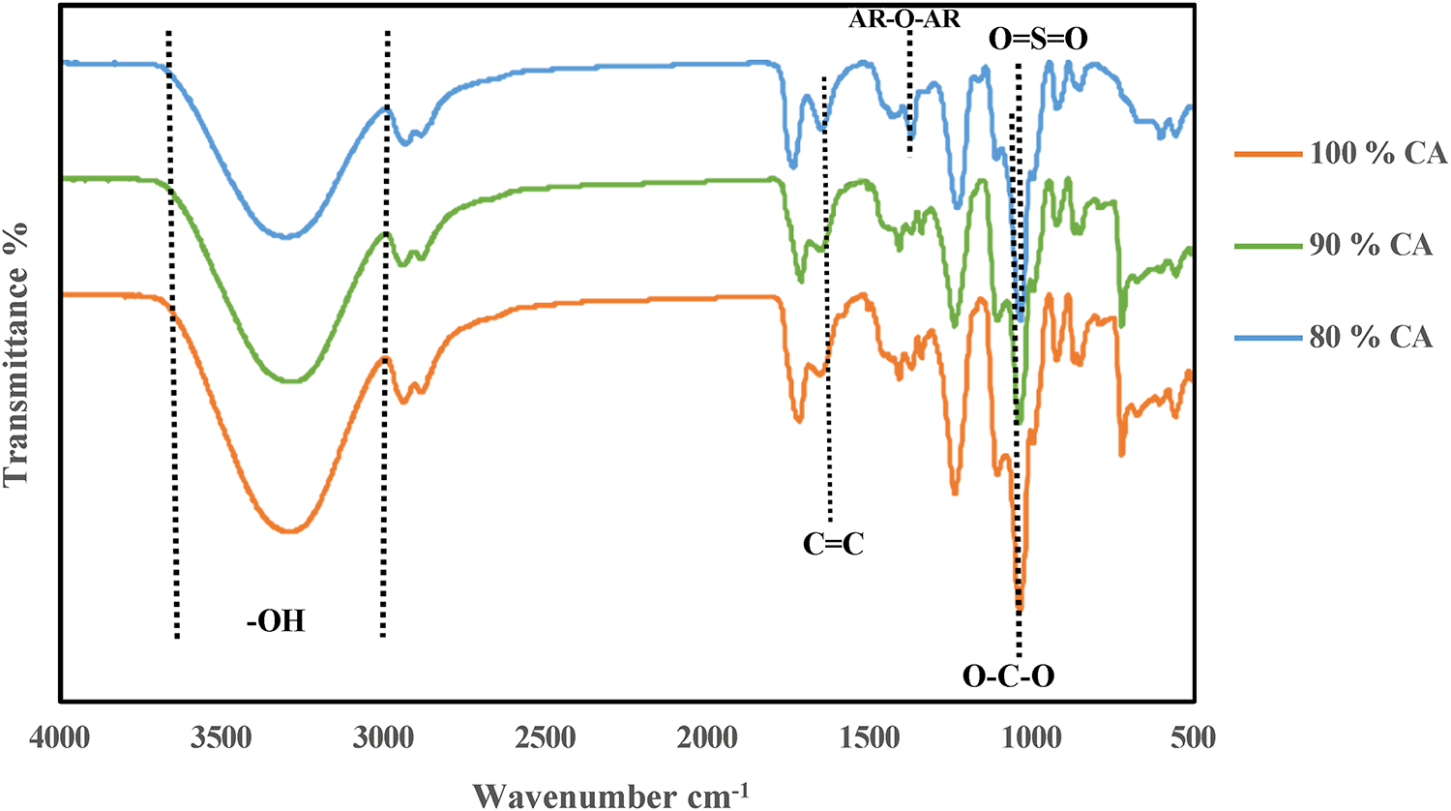
ATR-FTIR of prepared membranes at different polymer blend ratios.

Figure 2 depicts the interactions between CA and PS at different ratios (90/10 and 80/20) and its comparison with pure CA (100) through the investigation of FTIR spectroscopy for the three samples.

For the CA membrane, a broad band observed between 3200 and 3650 cm^−1^ was associated with the stretching of hydroxyl (^–^OH) groups. Additionally, the absorption band in the range of 2700 to 2900 cm^−1^ was attributed to the stretching vibrations of ^–^CH_2_ groups^70^. The ether linkage (C–O–C) appeared at 1065 cm^−1^, which indicated the glycosidic units^71^. In addition there are many waves numbers was observed at 2939 cm^−1^, 1730 cm^−1^, 1374 cm^−1^, 1250 cm^−1^ which related to CH_2_, C=O, CH_3_C=O and C–O stretching as confirmed by^72–74^.

The spectra of CA/PS blend membranes indicated that the intensity of the broad band of OH for pure CA is decreased with increasing polysulfone content 0/100, 10/90, 20/80 due to the formation of hydrogen bond between S=O and OH of cellulose acetate.

Additionally, the stretching frequency of the C–O bond in pristine CA, which is typically observed at 1010 cm^−1^, is shifted to 1045 cm^−1^ and 1037 cm^−1^ in CA/PS 90/10 and CA/PS (80/20) blend membranes, respectively. This can be attributed to the presence of symmetric S=O that appears at 1160 cm^−1^. Also, the intensity of CH₃– C=O is slightly increased due to its overlapping with asymmetric S=O that appears at 1370 cm^−1^, as agreed with Douna et al.^62^. In addition, the presence of a small peak appears at 1650 cm^−1^; its intensity increases with increasing PS concentrations as the presence of C=C of the aromatic benzene ring. The observed shifts in the stretching frequencies of CA/PS indicates the formation of hydrogen bond between the ^-^S=O^−^ group of PS and the ^–^OH group of CA at 3200 cm^−1^, this confirmed that the interaction is physically as confirmed by Jayalakshmi et al.^75^ and Douna et al.^62^.

#### Physical properties of membranes prepared at different ratios of CA/PS polymer blend

##### Membranes surface morphology

**Fig. 3:**
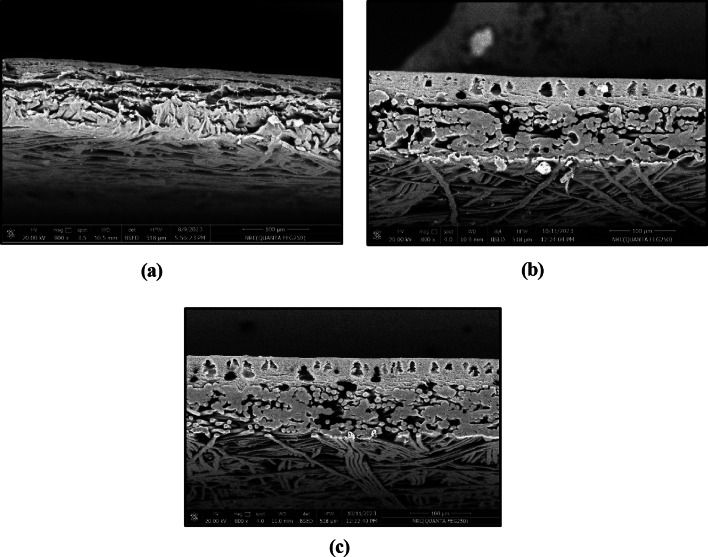
Surface morphology of the FO membrane polymer blend, (**a**) CA/PS: 100/0, (**b**) CA/PS: 90/10 and (**c**) CA/PS: 80/20.

Figure 3 represents cross-sectional images of the prepared membranes with two different blend ratios and compares them with the pure CA membrane. It is shown that 100% CA membrane is a symmetric, spongy, porous structure with a thick and dense selective layer (Fig. 3a). While the FO polymer blend membranes are asymmetric, as confirmed by the presence of microvoids surrounded by dense pores (Fig. 3b,c). These microvoids increase and become wider with increasing PS content from 10 to 20%. Moreover, the dense layer at the top surface decreases with increasing the blend ratio from 90/10 to 80/20.

##### Hydrophilicity, porosity and tensile strength of membranes

The hydrophilicity of CA and CA/PS blend membranes prepared at ratios of 100%, 90/10, and 80/20 was determined according to the value of the contact angle. A smaller contact angle indicates a higher degree of hydrophilicity in membranes, which were found to be 22.0° for 100% CA, 23.6° for CA/PS 90/10, and 25.9° for CA/PS 80/20. The physical properties of the produced membranes have been systematically analyzed and documented in Table 1 as well as commercial cellulosic FO membrane CA-NW 100^66^.

**Table 1:** Physical properties of prepared membranes at different polymer blend ratios.

Membrane blend ratio (CA/PS)	Water up take rate (%)	Porosity (%)	Contact angle	Tensile strength (MPa)	Thickness (µm)	Tortuosity (τ)	S (µm)
100/0	84	80.9	22^o^	79	105	1.75	227
90/10	81.9	78.5	23.6^o^	79	122	1.88	292
80/20	78.5	76.76	25.90°	79	134	1.98	345
CA-NW 100	67	56	64	9.5	125	3.7	825

It is demonstrated that the water content and porosity of the membrane exhibited a gradual decline as the polystyrene (PS) content in the polymer blend membrane increased from 10% to 20%. While the high hydrophilicity of the pure CA membrane was attributed to immediate phase de-mixing between the solvent (NMP) and the coagulant (distilled water), which is mainly the reason for the great number of pores and correspondingly the membrane water content^76^.

### FO membrane performance

#### FO membrane performance at different ratios of CA/PS polymer blend

The effect of various polymer blend concentrations on the membrane performance is shown in Table 2.

**Table 2:** Effect of polymer blend concentrations on FO membrane performance.

Membrane blend ratio (CA/PS)	Flux (LMH)	Reverse salt flux (mol/m^2^h)
100/0	28	0.18
90/10	110.67	1
80/20	134.67	2

In spite of the increasing PS content decreasing the membrane blend hydrophilicity, it was found that the water flux of the prepared FO membrane was increased. This improvement can be attributed to the role of PS in decreasing the interconnectivity between the dense pores of the CA membrane, which leads to the microvoids formation and, in turn, increases permeability. These microvoids increase and become wider with increasing PS content from 10 to 20% in the presence of PEG as a pore-forming agent^77^.

On the other hand, increasing the polysulfone content over 10%: a significant increase in the reverse salt flux was observed; this can be ascribed to the development of greater surface pore size, which was observed as a result of the average pore radius; this is in good agreement with Bagheripour et al.^78^. So, the optimum CA/PS ratio was chosen to be about 90/10 of the total polymer blend.

#### Effect of DS concentration on FO membrane performance

**Fig. 4:**
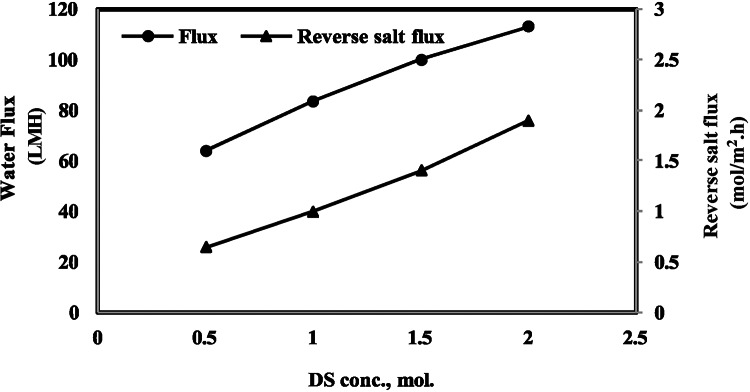
Effect of DS concentrations on water flux for prepared FO membrane at optimum polymer blend ratio of 90/10.

The FO performances for the prepared membrane at the optimum polymer blend ratio of 90/10 were tested for saline water desalination using NaCl draw solution at different concentrations. Figure 4 shows the change of water flux and reverse water flux with DS concentrations ranging from 0.5 to 2 M NaCl, and the feed solution is distilled water. Certainly, high performance was obtained, where the water flux and reverse salt flux reached 150.67 LMH and 2 mol/m^2^h, respectively, at 2 M NaCl DS.

The performance of the optimum membrane in terms of water flux is compared with some recent reported values in the literature (Table 3); it is obvious that the prepared membrane has a superior performance over other investigated synthesized membranes, except for Sun et al.^79^, which attained the highest flux in the literature (66.3 LMH) by using a prepared polyethylene-supported thin-film composite FO membrane under operating conditions similar to those in the current study (DI FS and 0.5 M NaCl DS). It is clear that, in the current study, the flux (85 LMH) is much higher than their result, but taking into consideration the drawbacks of TFC membranes, including higher membrane fouling tendency and higher manufacturing and maintenance costs, in addition to the capability of subjecting the polyamide active layer to chlorine attack^42,50,51^. So, it can be concluded that the CA/PS polymer blend FO membrane in this study has the best performance.

**Table 3:** Comparison of membrane performance with literature.

Membrane type	Feed solution	Draw solution	Water flux (LMH)	References
CA/PS blend (ratio 90/10)	Distilled water	0.5 M–2 M NaCl	85–150	This study
CA multi-layer membrane using PDA and Cs NP through Layer by layer coating	DI	2 M NaCl	21.23	Askar et al., 2024^80^
CTA/CA /PVP	DI	4 M NaCl	5.8	Idris et al., 2024^81^
CTA pattern (10% CTA MWt 300 dissolved in DMSO	DI	2 M NaCl	30	Ilyas et al., 2022^82^
Polyethylene supported thin-film composite	DI	0.5 M NaCl	66.3	Sun et al., 2022^79^
CA 7% + 0.25% ZnO NPs using Acetone as solvent	1000 mg/L NaCl	10,000 mg/L MgSO4	26.57	El-Noss et al., 2020^83^
CA modified with PVA and PDA	Distilled water	2 M NaCl	16.72	Song et al., 2018^84^
CTA/CA	Distilled water	1 M NaCl	10.39	Nguye et al., 2013^85^

### Application for brine concentration

#### Effect of different draw solution molar ratio on water flux

**Fig. 5:**
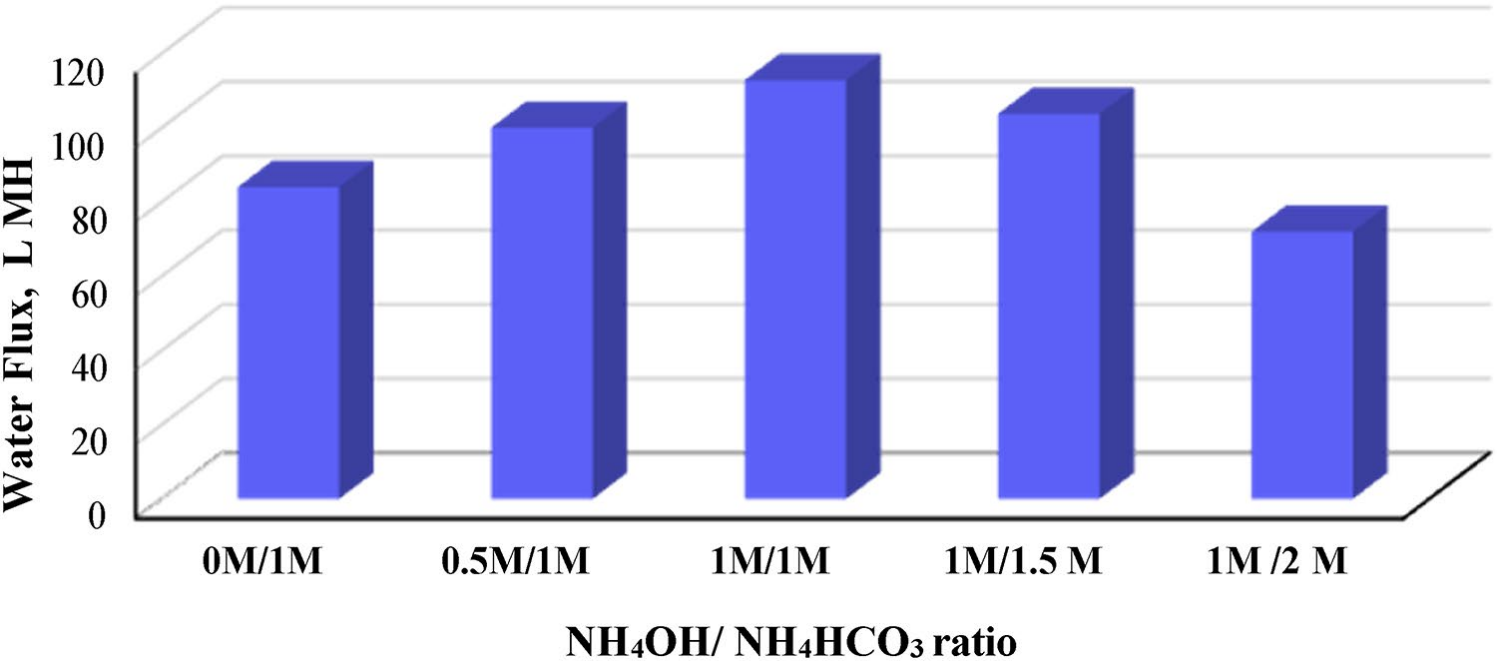
Effect of ammonium hydroxide/ammonium bicarbonate mixture ratio as draw solution on water flux of brine concentration.

**Fig. 6:**
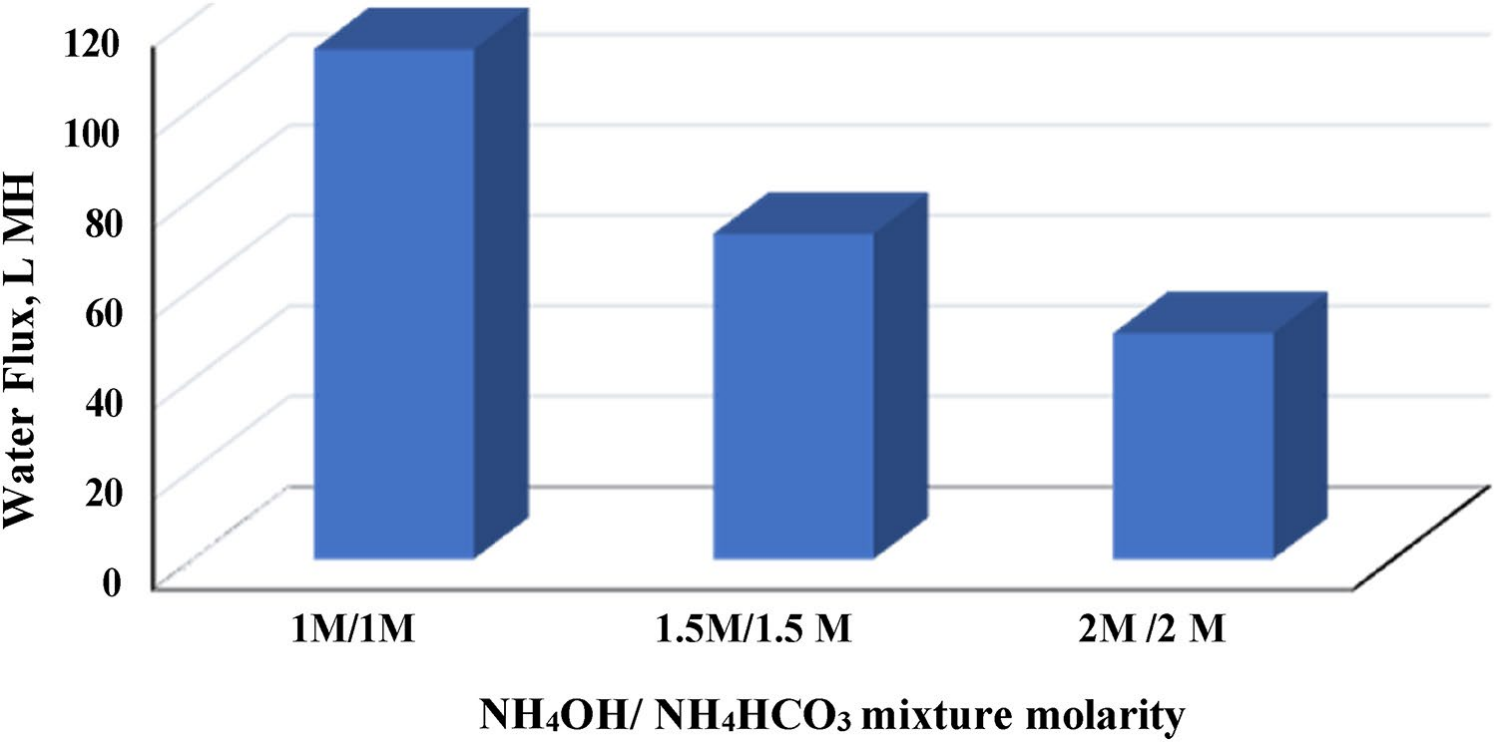
Effect of ammonium hydroxide/ammonium bicarbonate mixture molarity (at optimum ratio 1:1) as draw solution on water flux of brine concentration.

The performance of prepared FO membrane at optimum polymer blend ratio of 90/10 as membrane brine concentrator was studied by using a mixture of ammonium bicarbonate and ammonium hydroxide at different molar ratio. The feed solution is a synthetic brine consists of a commercial NaCl solution at concentration 65 mg/L. Figure 5 demonstrates the change in FO membrane performance in terms of water flux against different NH_4_OH/NH_4_HCO_3_ molar ratios (0 M/1 M, 0.5 M/1 M, 1 M/1 M, 1 M/1.5 M and 1 M/2 M). It is clear that addition of NH_4_OH increasing the flux from 84 LMH for 0 M/1 M NH_4_OH/NH_4_HCO_3_ to about 100 and 113 LMH at ratios of 0.5 M/1 M and 1 M/1 M respectively. By further increasing the DS concentration to 1 M/1.5 M, a slightly decrease in water flux to 78 LMH was observed, then it decreases sharply to 72 LMH by increasing molar ratio to 1 M/2 M. On the other hand, the effect of NH_4_OH/NH_4_HCO_3_ mixture molarity at optimum ratio 1:1 as DS on water flux of brine concentration is illustrated in Fig. 6. It was obvious that increasing mixture concentration decreased the flux sharply from 85 LMH at 1 M/1 M DS concentration to 54 and 37.5 LMH at 1.5 M/1.5 M and 2 M/2 M respectively.

While, increasing water flux with increasing DS concentration is well known behavior in FO process which explained by increasing of driving force across the membrane^67,86,87^, the phenomena of its decreasing by further increasing in DS concentration, was explained by McCutcheon et al.^88^. They demonstrated that higher DS concentration increases the harshness of dilutive concentration polarization CP, subsequently the high draw solution concentrations yielding high water flux rates. The integration between the concentrative and dilutive external CP phenomena is the reason for the reduction of the effective osmotic pressure driving force.

(Table 3). Comparing with membranes brine concentrator in literature, Table 4 illustrate the most recent studies on the brine concentration using forward osmosis system with different types of membrane. It was found that the prepared membrane attained much higher performance. For examples; McCutcheon et al.^88^, used HTI commercial FO membrane for concentrating of 1 M NaCl synthetic solution using NH_4_OH/NH_4_HCO_3_ mixture draw solution at 4.5 molar ratio. The highest water flux obtained was about 16 LMH. Tang and Ng^36^, compared three types of membranes: HTI FO membrane, the dense selective layers of cellulose acetate asymmetric RO and the dense selective layer of polyamide composite membrane for the concentration of synthetic NaCl brine solutions by using fructose up to 5 M concentration as draw solution. The better performance was achieved by cellulose acetate asymmetric RO membrane which gave a water flux of 15.0 LMH. Lee et al.^89^, used woven HTI FO membrane for concentrating real RO brine using 3 M MgCl_2_ as DS, the maximum water flux attained was 20 LMH. Recently, commercial FO membrane was used to concentrate brine from nanofiltration membrane, and the maximum flux reaches around 29 LMH^97^. Also, Z-nano Aquaporin membrane was investigated for the concentration of RO brine, the FO flux ranges between 29–35 LMH.

**Table 4:** Comparison of membrane brin concentrator flux with literature.

Membrane type	Feed solution	Draw solution	Water flux (LMH)	References
CA/PS blend (ratio 90/10)	Na Cl 65 mg/L	NH_4_OH/NH_4_HCO_3_	84–113	This study
CTA-Woven membrane (HTI)	NaCl	Amm.Bicar. + amm.hydroxide in fixed ratio		McCutcheon et al., 2006^88^
	1 M	3	8.15	
	1 M	4.5	15.96	
	2 M	4.5	3.9	
	2 M	6	7.3	
CTA	1 M NaCl	5 M Fructose	15	Tang and Ng, 2008^36^
	1.5 M	5–6 M	8	
CTA, polyamide (PA)]	Synthetic wastewater	NaCl (35–200 g L^−1^)	CTA (0.032–0.56) Poly Amide (0.419–2.785)	Abdulwahab et al., 2013^90^
CTA-W	RO brine	NaCl (2–3)M	9.1–13.5	Jamil et al., 2015^91^
CTA-W	RO brine	3 M MgCl_2_	20	Lee et al., 2016^89^
CTA-NW (HTI)	RO brine as synthetic solution	100 g/L NaCl	3.46	Eusebio et al., 2016^92^
CTA	High salinity	NH_3_-CO_2_	1	Al-Furaiji, 2016^93^
	PW	and MgCl_2_	4.5	
CTA and TFCPA	PW	NaCl	3	Coday et al., 2016^94^
TFC –ES	Brine after extraction of potassium	1-NaCl	7.4–6.8	Li et al., 2018^95^
CTA-NW (HTI)		2-MgCl_2_ precipitated from salt lake brine	9.3–8.5	
Commercial PA FO membrane from proifera grade	Synthetic brine using 65 g NaCl in both	1 M Ammonium sulfate		El Zayat et al., 2021^96^
	Reagent grade		11.69	
	Industrial		8..63	
Commercial FO membrane	Brine after nano filtration membrane separation	Hyper saline NaCl solution	18.28	Zhilu Li et al., 2024^97^
		and Salt lake brine after K potassium precipitated	29.78	
Commercial FO –TFC membrane	Thermal evaporator brine	Amm. Bicarbonate 3 M	6	Sitabule et al., 2023^98^
Z-nano Aquaporin membrane	RO brine	26% NaCl	29.7–35.7	Bhadrachari et al., 2023^99^

#### Membrane fouling tendency

**Fig. 7:**
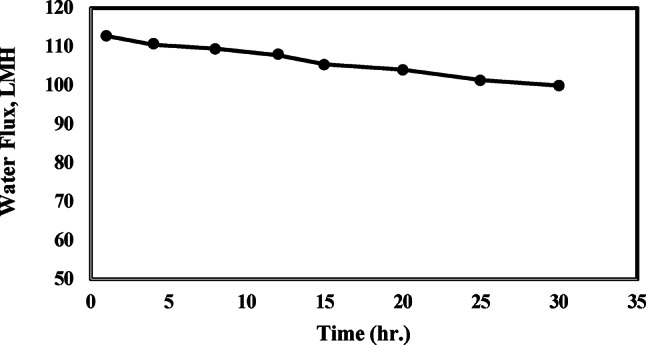
Effect of time on FO performane for brine concentration at constant Ammonium hydroxide/ammonium bicarbonate mixture ratio (optimum ratio 1:1) as draw solution.

The membrane fouling tendency of the optimum FO membrane was examined for brine concentration using a constant NH_4_OH/NH_4_HCO_3_ draw solution with a mixture ratio of 1:1 as shown in Fig. 7. It was observed that the flux retained a consistently high value with time, where it decreases slightly from about 113.3 LMH at 1 h to 100 LMH after 30 h. This may be attributed to the low fouling tendency of the prepared FO membrane that was confirmed with its physical characterization, which includes low tortuosity, a low S structure, and high porosity compared to the commercial cellulosic FO membrane CA-NW 100, as mentioned previously in Table 1.

**Fig. 8:**
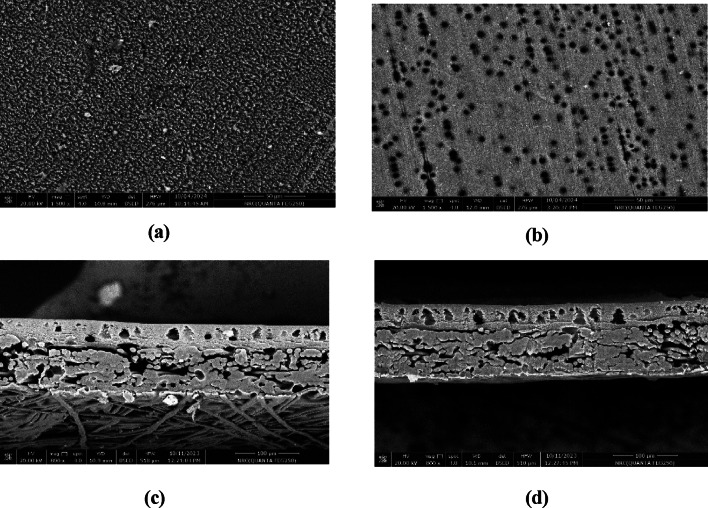
Surface morphology and cross section of virgin (**a**,**c**) and fouled (**b**,**d**) membrane.

Figure 8 shows membrane surface morphology (a, b) and cross-section (c, d) of virgin and fouled membranes. It was observed that there is no foulants in the pores of the tested membrane as compared with the virgin one. In addition, the thickness of the investigated membrane is lower than the virgin membrane, this is attributed to membrane compression during the application.

## Conclusions

In conclusion, this research presents a novel cellulosic FO membrane developed through the blending of CA and PS, aimed at improving desalination and brine concentration processes. Through detailed characterization methods, including SEM, FTIR, and various physical property assessments, the study established that the membrane’s morphology and properties are significantly influenced by the CA/PS blend ratio. The SEM analysis demonstrated that blending with PS resulted in asymmetric membrane structures with enhanced microvoid formation, which contributes to increased permeability. The optimal CA/PS blend ratio of 90/10 demonstrated the best overall performance, achieving a maximum of 150 and 113 LMH for desalination and brine concentration, respectively. Although higher PS content slightly reduced the membrane’s hydrophilicity, the resultant pore structure improvements effectively elevated water flux. Additionally, the membrane exhibited a low fouling tendency during brine concentration, sustaining high performance over prolonged use. Elemental analysis and SEM observations of the fouled membrane indicated minimal fouling, attributed to its physical characteristics such as low tortuosity and high porosity. Overall, the study underscores the effectiveness of the CA/PS blend membrane in forward osmosis applications, offering a promising and sustainable approach for water desalination and brine management.

## Data Availability

All data generated or analyzed during this study are included in this published article and available from the corresponding author on reasonable request.
