# Author Correction: Superior forward osmosis cellulosic membrane for water desalination and brine concentration

**DOI:** 10.1038/s41598-025-11479-9

**Published:** 2025-07-25

**Authors:** Rania Sabry, Hanaa M. Ali, Sahar S. Ali, Hanaa Gadallah

**Affiliations:** Chemical Engineering Department, Engineering and Renewable Energy Research Institute, National Research Centre, 33 El-Bohouth St. (Former El-Tahrir St.), PO Box 12622, DokkiGiza, Egypt

Correction to: *Scientific Reports* 10.1038/s41598-025-00141-z, published online 14 May 2025

The original version of this Article contained a typographical error in Eq. 3, where “**^2**” was omitted.

$$\tau = \left( {2 - P} \right)/P$$

now reads:

$$\tau = {\left( {2 - P} \right)^2}/P$$

The original Article has been corrected.

